# Accumulation of Dystrophin-Positive Muscle Fibers and Improvement of Neuromuscular Junctions in mdx Mouse Muscles after Bone Marrow Transplantation under Different Conditions

**DOI:** 10.3390/ijms24108892

**Published:** 2023-05-17

**Authors:** Anastasiia V. Sokolova, Alisa P. Domnina, Viacheslav M. Mikhailov

**Affiliations:** Institute of Cytology, Russian Academy of Sciences, 194064 Saint-Petersburg, Russia

**Keywords:** muscular dystrophy, dystrophin, mdx mice, bone marrow cell, neuromuscular junctions

## Abstract

Duchenne muscular dystrophy (DMD) is a severe muscular disorder caused by mutations in the dystrophin gene. It leads to respiratory and cardiac failure and premature death at a young age. Although recent studies have greatly deepened the understanding of the primary and secondary pathogenetic mechanisms of DMD, an effective treatment remains elusive. In recent decades, stem cells have emerged as a novel therapeutic product for a variety of diseases. In this study, we investigated nonmyeloablative bone marrow cell (BMC) transplantation as a method of cell therapy for DMD in an mdx mouse model. By using BMC transplantation from GFP-positive mice, we confirmed that BMCs participate in the muscle restoration of mdx mice. We analyzed both syngeneic and allogeneic BMC transplantation under different conditions. Our data indicated that 3 Gy X-ray irradiation with subsequent BMC transplantation improved dystrophin synthesis and the structure of striated muscle fibers (SMFs) in mdx mice as well as decreasing the death rate of SMFs. In addition, we observed the normalization of neuromuscular junctions (NMJs) in mdx mice after nonmyeloablative BMC transplantation. In conclusion, we demonstrated that nonmyeloablative BMC transplantation could be considered a method for DMD treatment.

## 1. Introduction

Duchenne muscular dystrophy or Duchenne myodystrophy (DMD; OMIM #310200) is an X-linked recessive muscular dystrophy. It is one of the most clinically severe and common muscular dystrophies in humans [1,2,3,4]. DMD affects approximately 1 in 3500–6300 live-born boys worldwide, causing a severe loss of muscle mass and function and leading to death in the late teenage years due to respiratory or cardiac failure [5,6]. Furthermore, DMD is associated with various mutations in the *DMD* gene that encodes the protein dystrophin. The dystrophin complex forms a mechanically strong link between the sarcolemma and the costameric cytoskeleton through interacting with γ-actin filaments [7,8]. As a result of *DMD* mutations, which cause a lack of functional dystrophin, the cell membrane surrounding skeletal muscle fibers—sarcolemma—becomes fragile and is damaged during skeletal muscle contraction, which leads to intrinsic myofiber necrosis (or myonecrosis). Cyclical myofiber degeneration and regeneration are the reason for the repair failure and replacement of muscle with fibrotic and adipose tissue. The loss of muscle fibers leads to progressive weakness, loss of ambulation, and premature death.

Various animal models of DMD, such as *mdx* mice, golden retriever muscular dystrophy (GRMD), hypertrophic feline muscular dystrophy, and genetically modified animals have been described [9]. One of the most widely used laboratory models of DMD is the *mdx* mouse model. This strain appeared in the early 1980s in a colony of C57BL/10 mice as the result of a spontaneous nonsense mutation in the dystrophin gene [10,11]. *mdx* mice are characterized by the absence or very low levels (revertant fibers) of dystrophin and a high rate of muscle fiber death. While significant differences exist in the course of dystrophy in *mdx* mice and humans with DMD, *mdx* mice remain a widespread model of this disease.

However, although numerous studies have been conducted, an effective treatment for DMD remains elusive. Currently, gene therapy methods and cell therapy studies are being developed with the aim of finding methods of treating DMD [5,12,13,14,15]. Significant progress has been made in the development of exon skipping methods, but numerous difficulties remain. Antisense oligonucleotide (AO)-mediated exon skipping is a mutation-specific approach that is currently only applied to deletion mutations [14]. Furthermore, the delivery of AOs has always been a significant barrier to their broad clinical application [16]. Moreover, currently approved AOs are quite expensive. Given all of the limitations of gene therapy methods, stem cell transplantation is now considered a promising approach [12]. Cells of different origins are capable of myogenic differentiation, such as satellite cells [17,18], mesoangioblasts [19,20], pericytes [21], side population cells [22], muscle-derived stem cells [23,24,25], blood- or muscle-derived CD133(+) stem cells [26,27], induced pluripotent stem cells [28], dystrophin-expressing chimeric cells of myoblast origin [29,30], and bone marrow stem cells (BMCs) [23,31]. A potential cell therapy method for treating DMD is bone marrow transplantation. Since BMCs have been demonstrated to be involved in muscle regeneration [32,33], it has been assumed that donor cells, after bone marrow replacement, would participate in the regeneration of the recipient’s muscles; thus, nuclei with the normal *DMD* gene would enter the muscle fibers, in which dystrophin would be expressed. Numerous studies on stem cell transplantation have also been conducted on *mdx* mice. However, BMC transplantation experiments on *mdx* mice after total body irradiation by ionizing radiation—at doses that do not allow autologous hematologic recovery (myeloablative transplantation)—have failed to achieve a significant increase in the proportion of dystrophin-positive fibers [23,34,35,36]. It seems that the incorporation of normal nuclei into striated muscle fibers (SMFs) alone is not sufficient for activating the expression of muscle-specific genes [37]. It is possible that the low dystrophin increases in *mdx* mice SMFs after BMC transplantation that follows lethal irradiation is associated with the negative influence of ionizing radiation on muscle tissue [38].

In this study, we tested nonmyeloablative syngeneic and allogeneic bone marrow transplantation while reducing the dose of ionizing radiation as a method for myodystrophy correction in *mdx* mice.

## 2. Results

### 2.1. mdx Mouse Muscle Histology

**Figure 1:**
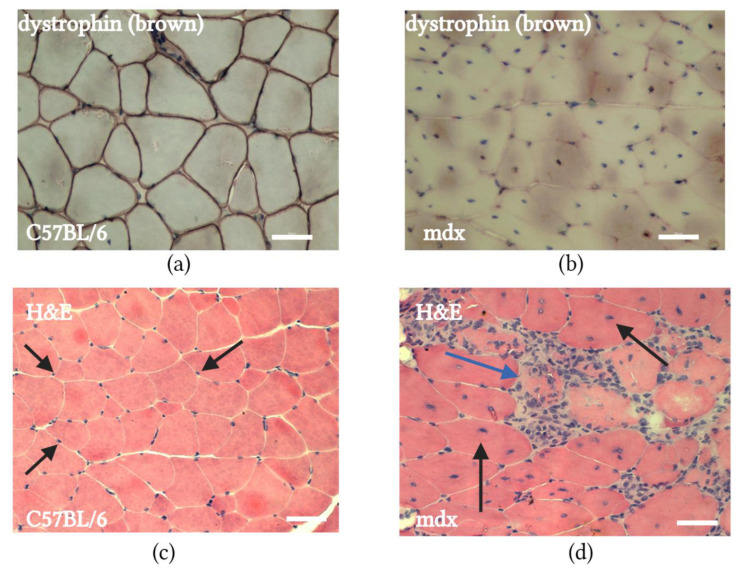
Histological sections of quadriceps femoris muscles of normal C57BL/6 mice and *mdx* mice: (**a**) immunohistochemical staining of SMFs from normal C57BL/6 mice for dystrophin; scale bar 50 µm; (**b**) immunohistochemical staining of SMFs from *mdx* mice for dystrophin; dystrophin in *mdx* mouse muscle was missing in all SMFs; scale bar 50 µm; (**c**) H&E staining of SMFs from normal C57BL/6 mice; nuclei in the SMFs of C57BL/6 mice located at the periphery of the fiber (black arrows); scale bar 50 µm; and (**d**) H&E staining of SMFs from *mdx* mice. Centrally located nuclei are presented in the SMFs of *mdx* mice (black arrows), while the blue arrow demonstrates necrosis with inflammatory cell infiltration; scale bar 50 µm.

Histologically, the muscle tissue of *mdx* mice is characterized by the absence of dystrophin in muscle fibers due to mutations in the *DMD* gene [39]. Figure 1b demonstrates the absence of dystrophin-positive SMFs in *mdx* mice compared with syngeneic normal C57BL/6 mice (Figure 1a). One can also observe that in *mdx* mice, unlike C57BL/6 mice, SMFs differ in area and the nuclei are localized mainly in the center of the muscle fiber. The centrally located nuclei in SMFs were determined to be discontinued differentiation into muscular fibers at the myotube stage. This was used as an indicator of the differentiation of SMFs. In addition, SMF necrosis and inflammatory cell infiltration were clearly detected in *mdx* mouse muscle (Figure 1d). Moreover, mdx muscles contain more fibrotic tissue relative to C57BL/6 (Supplementary Figure S1), which corresponds with the literature data [40]. In mdx mice, the area of muscle fibers has been shown to vary much more than in C57BL/6 mice. However, we did not find significant differences in the mean cross-sectional area (CSA) of muscle fibers in normal C57BL/6 mice (3765 ± 260 μm^2^) and mdx mice (3159 ± 348 μm^2^), which is consistent with some literature data [40]. The frequency distribution of muscle fiber in the CSA of mdx mice showed muscle fibers with varied sizes, with a predominance of muscle fibers with a small area, which is associated with the presence of regenerating fibers. Moreover, the muscle of mdx mice contained SMFs with a large CSA, resulting from hypertrophy of SMFs (Supplementary Figure S2).

### 2.2. mdx Mouse Muscle Contained GFP-Positive Cells and SMFs after Transplantation of BMCs from Transgenic GFP-Expressing C57BL/6 Mice

**Figure 2:**
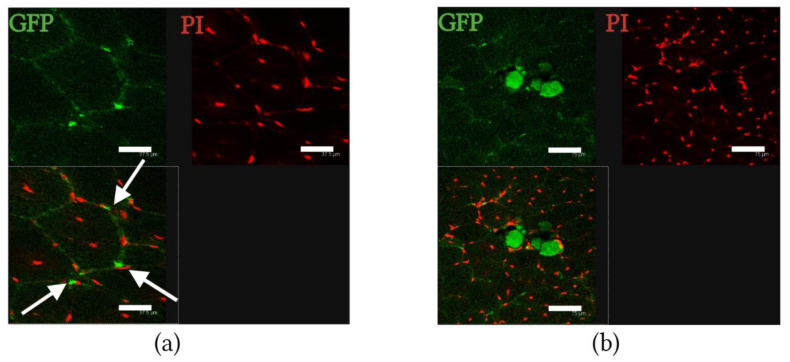
Histological sections of quadriceps femoris muscles of *mdx* mice 6 months after transplantation of GFP-positive BMCs: (**a**) GFP-positive cells (green, white arrows) had survived and were present in the quadriceps femoris muscles of *mdx* mice. Nuclei were stained with propidium iodide (red); scale bar 37.5 µm; and (**b**) GFP-positive SMFs (green) were observed in *mdx* mice quadriceps femoris muscles. Nuclei were stained with propidium iodide (red); scale bar 75 µm.

We transplanted GFP-positive BMCs into *mdx* mice after X-ray irradiation with 3 Gy to ensure that the donors’ BMCs could survive in the recipient and be involved in muscle regeneration. We observed both individual GFP-positive cells between SMFs and GFP-positive SMFs in the muscles of *mdx* mice 6 months after transplantation (Figure 2). The proportion of GFP-positive SMFs in the mdx mice was (0.8 ± 0.3)%.

### 2.3. Transplantation of BMCs from C57BL/6 Mice Restored SMFs in mdx Mice

**Figure 3:**
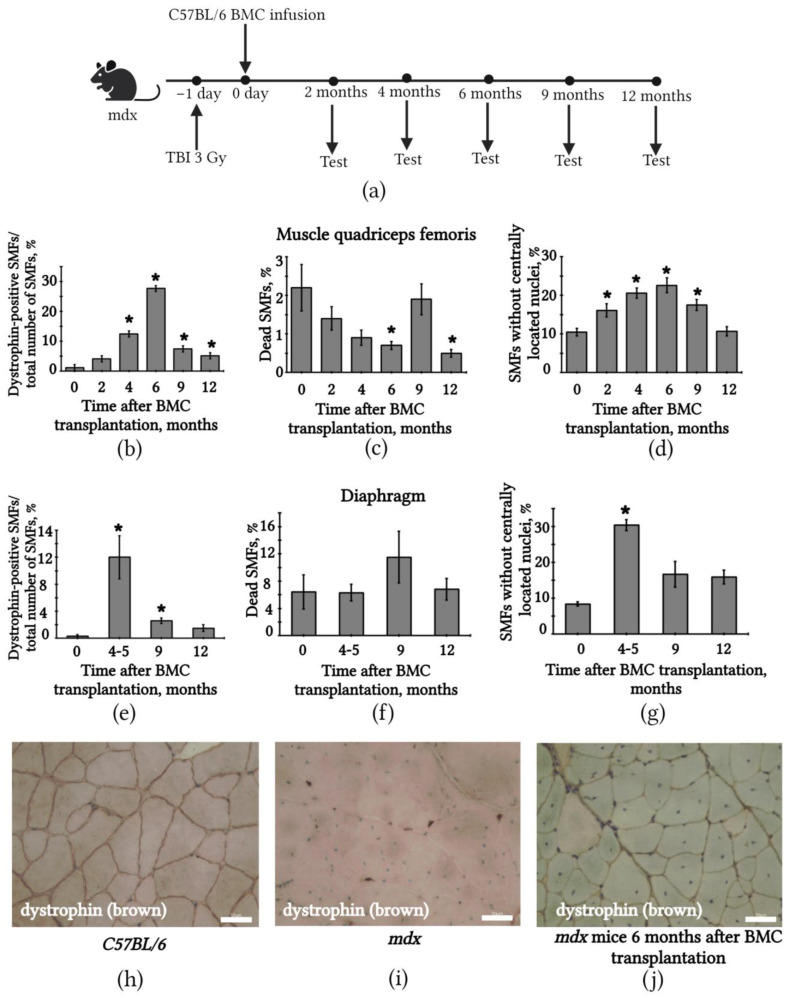
Nonmyeloablative intravenous transplantation of C57BL/6 BMCs led to SMF restoration in *mdx* mouse muscles. (**a**) Scheme of experiments, TBI—total body irradiation; (**b**) percent number of dystrophin-positive SMFs in *mdx* mouse quadriceps femoris muscles started to increase post-transplantation; (**c**) percentage of dead SMFs in the quadriceps femoris of *mdx* mice significantly decreased 6 months after BMC transplantation; (**d**) percent number of SMFs without centrally located nuclei in the quadriceps femoris of *mdx* mice started to increase 2 months after BMC transplantation; (**e**) percent number of dystrophin-positive SMFs in *mdx* mouse diaphragm muscles drastically increased 4–5 months after BMC transplantation; (**f**) percentage of dead SMFs in the diaphragm of *mdx* mice following BMC transplantation; (**g**) percent number of SMFs without centrally located nuclei in the diaphragm of *mdx* significantly increased 4–5 months after BMC transplantation; (**h**) immunohistochemical staining of SMFs from the quadriceps femoris of normal C57BL/6 mice for dystrophin (brown); nuclei in SMFs of C57BL/6 mice located at the periphery of the fiber (blue); scale bar 50 μm; (**i**) immunohistochemical staining of SMFs from the quadriceps femoris of *mdx* mice for dystrophin; dystrophin in *mdx* mouse muscle was missing in all SMFs; nuclei in the SMFs are centrally located (blue); scale bar 50 μm; and (**j**) immunohistochemical staining of SMFs from the quadriceps femoris of *mdx* mice 6 months after BMC transplantation; numerous dystrophin-positive SMFs are observed (brown); scale bar 50 μm. The results are means ± SD. * *p* < 0.05 vs. mdx (0 months after BMC transplantation).

We transplanted BMCs from C57BL/6 mice to X-ray *mdx* mice irradiated with 3 Gy (Figure 3a). It was observed that 6 months after intravenous transplantation, the number of dystrophin-positive SMFs in the quadriceps femoris had drastically increased (Figure 3b). Furthermore, the percentage of SMFs without centrally located nuclei had increased (Figure 3d), while the percentage of dead SMFs had decreased (Figure 3c) compared with the untreated group of *mdx* mice. Nine months after transplantation, the number of dystrophin-positive SMFs in the quadriceps muscles of the treated *mdx* mice had decreased to 7.4 ± 1.5%, and by 12 months, the decrease had continued to 5.1 ± 1.1% (Figure 3b). The percentage of dead SMFs also increased with time (Figure 3c). Six months after the transplantation of C57BL/6 BMC into the mdx mice, there was a significant decrease in the proportion of dead muscle fibers in the quadriceps muscles of mdx mice by three times compared to the untreated mdx mice, and after 12 months, by four times (Supplementary Figure S3). After BMC transplantation, we found a decrease in the proportion of connective tissue in muscle in mdx mice in histological section imaging (Supplementary Figure S1). In mdx mice, SMFs have been shown to be more heterogeneous in their CSA, with an increase in the proportion of muscle fibers with a smaller area compared to normal C57BL/6 animals. Six months after BMC transplantation, there was a decrease in muscle fibers with a small CSA, an absence of SMFs with a large CSA, and an increase in muscle fibers with a medium CSA, which approximates the CSA distribution in mdx mice after bone marrow cell transplantation to that of normal mice C57BL/6 (Supplementary Figure S2).

In the diaphragms of the *mdx* mice, the number of dystrophin-positive SMFs as well as the percentage of SMFs without centrally located nuclei significantly increased at 4 months after BMC transplantation, and then started to decrease (Figure 3e,g). Simultaneously, in the diaphragm, no significant decrease in the number of dead SMFs was observed (Figure 3f).

### 2.4. Changes in the Structure of Neuromuscular Junctions in mdx Mice after BMC Transplantation from C57BL/6 Mice

**Figure 4:**
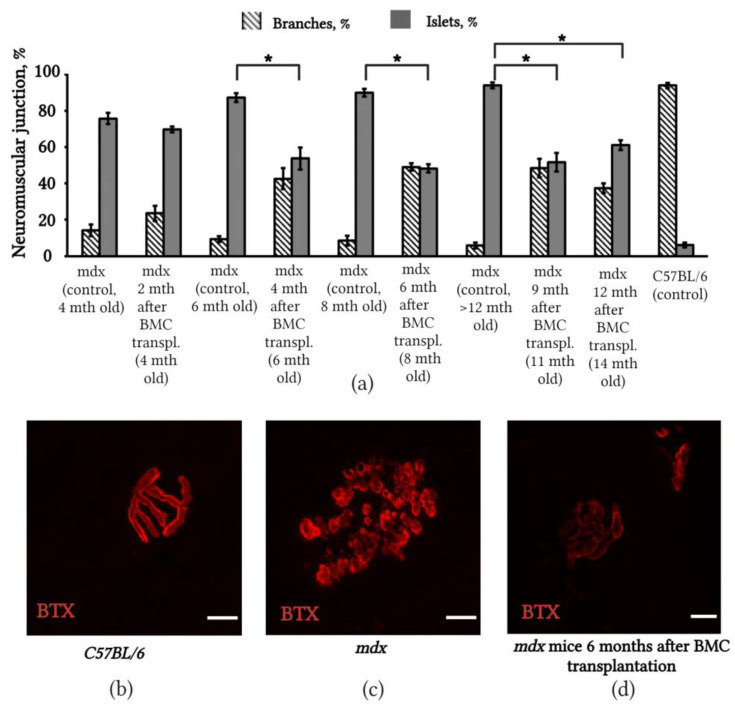
NMJs of *mdx* mice improved 4 months after C57BL/6 BMC transplantation. (**a**) Changes in NMJs’ structure in *mdx* mice diaphragms after BMC transplantation; *mdx* mice of the same age were used as controls; the number of NMJs with a normal NMJ structure (AChRs were distributed as branches) increased; (**b**) digital images of NMJs from the whole-mount diaphragm of C57BL/6; (**c**) *mdx* control mice and (**d**) *mdx* mice 6 months after C57BL/6 BMC transplantation. AChRs in NMJs were labeled with tetramethylrhodamine-α-bungarotoxin (red, BTX); scale bar 10 µm. The results are means ± SD. * *p* < 0.05.

We investigated the changes in the structure of NMJs after BMC transplantation to identify the correlation between the accumulation of dystrophin in SMFs and the restoration of the structure of NMJs in *mdx* mice. We found that in mature NMJs of C57BL/6 mice, acetylcholine receptors (AChRs) were organized in a pretzel-like structure that contained elongated, uniformly fluorescent branches. These branches formed continuous channels, which extended in several directions along the muscle fiber—similar to what is usually observed in normal muscles (Figure 4b). By contrast, in *mdx* mice, we observed that large AChR clusters broke down into smaller ones and took the form of individual islets, in which the peripheral part was intensely colored and the central part was dark (Figure 4c). In the diaphragms of *mdx* mice, the number of NMJs with a normal structure, in which AChRs were distributed as branches, had increased 6 months after transplantation (Figure 4c). This effect was maintained at both 9 and 12 months (Figure 4a).

### 2.5. Changes in SMFs of mdx Mice after BMC Transplantation from C3HA Mice

**Figure 5:**
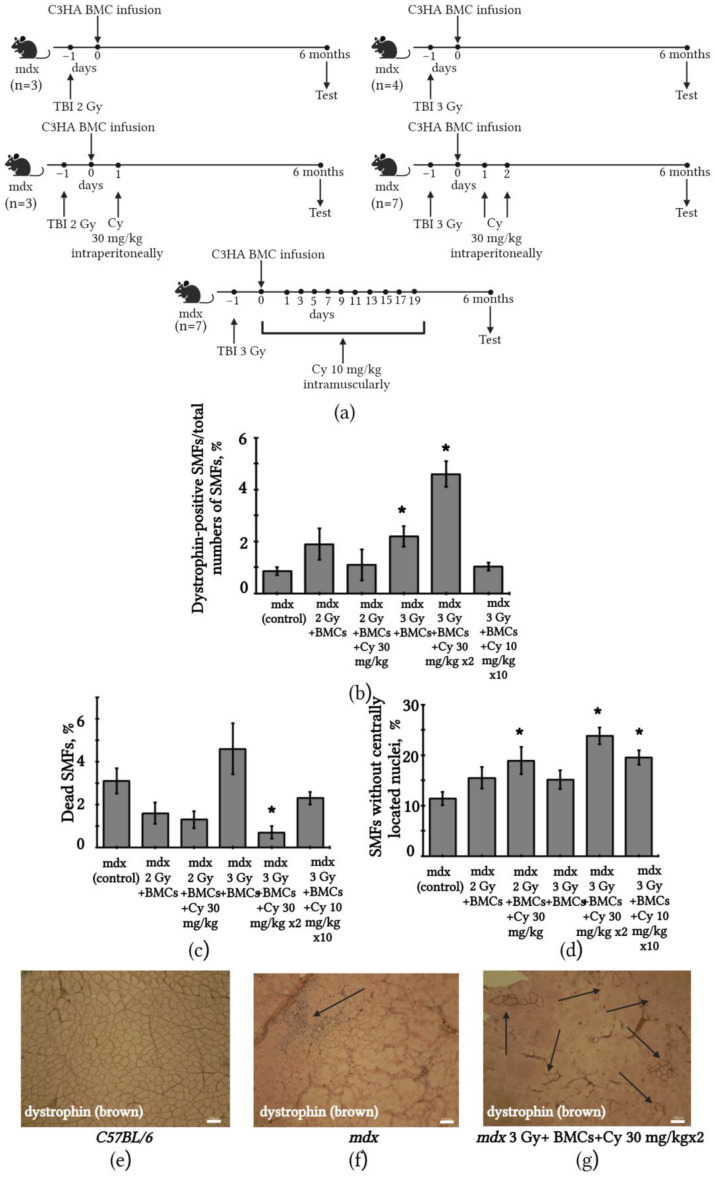
Nonmyeloablative intravenous transplantation of allogeneic C3HA BMCs led to SMF restoration in *mdx* mouse muscles. (**a**) Scheme of experiments, TBI—total body irradiation; (**b**) percent number of dystrophin-positive SMFs in the quadriceps femoris increased in treated *mdx* mice following X-ray irradiation of 3 Gy before transplantation with and without Endoxan injection in the post-transplantation period; (**c**) the percentage of dead SMFs in the quadriceps femoris of *mdx* mice significantly decreased in the group treated with X-ray irradiation of 3 Gy before transplantation with an Endoxan injection in the post-transplantation period; (**d**) an increase in the percent number of SMFs without centrally located nuclei in the quadriceps femoris of *mdx* mice was observed in all groups of animals with Endoxan administration; (**e**) immunohistochemical staining of SMFs from the quadriceps femoris of normal C57BL/6 mice for dystrophin (brown); scale bar 100 μm; (**f**) immunohistochemical staining of SMFs from the quadriceps femoris of *mdx* mice for dystrophin; dystrophin in *mdx* mouse muscles was missing in all SMFs; SMF necrosis with inflammatory cell infiltration was detected (black arrow); scale bar 100 μm; and (**g**) immunohistochemical staining of SMFs from the quadriceps femoris of *mdx* mice after C3HA BMC transplantation; numerous focuses of dystrophin-positive SMFs were observed (black arrows); scale bar 100 μm. The results are means ± SD. * *p* < 0.05 vs. mdx (control).

In previous experiments, we observed SMFs’ restoration in *mdx* mice after syngeneic BMC transplantation from C57BL/6 mice. In the next series of experiments, we aimed to verify the effectiveness of the allogeneic transplantation of BMCs from C3HA mice for SMF restoration in *mdx* mice. In the previous experiments, the maximum SMF recovery was observed 6 months after BMC transplantation. Therefore, we used this time period in the next round of experiments. To reduce the immune response of the recipient due to the allogeneic origin of the transplanted BMCs post-transplantation, injections of cyclophosphamide were performed. We tested two doses of X-ray irradiation (2 and 3 Gy) as well as comparing the effectiveness of allogeneic BMCs with and without the administration of cyclophosphamide Endoxan injections in two doses (10 or 30 mg/kg; Figure 5a). The number of dystrophin-positive SMFs in *mdx* mice drastically increased in the group of animals treated with X-ray irradiation at a dose of 3 Gy before transplantation and Endoxan injection at a dose of 30 mg/kg for 2 days in the post-transplantation period. (Figure 5b). Moreover, in said experimental condition, the percentage of dead SMFs was the lowest (Figure 5c) and was decreased by four times compared to the untreated mdx mice (Supplementary Figure S3). Statistically significant differences in the number of dystrophin-positive SMFs were also observed with 3 Gy X-ray irradiation without Endoxan injections compared with untreated *mdx*. An increase in the number of SMFs without centrally located nuclei was observed in all groups of *mdx* mice with Endoxan administration (Figure 5d).

### 2.6. Changes in NMJs’ Structure in mdx Mice after BMC Transplantation from C3HA Mice

**Figure 6:**
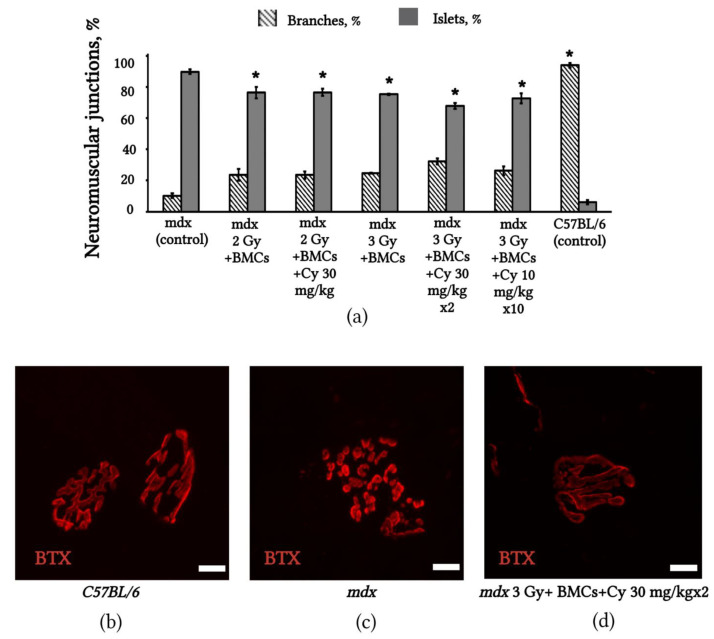
NMJs of *mdx* mice improved after allogeneic C3HA BMC transplantation: (**a**) Changes in NMJs’ structure in *mdx* mouse diaphragms after C3HA BMC transplantation under different conditions; the number of NMJs with a normal structure (AChRs distributed as branches) significantly increased in all groups of treated *mdx* mice; (**b**) digital images of NMJs from a whole-mount diaphragm of C57BL/6, (**c**) *mdx* control mice and (**d**) *mdx* mice after allogeneic C3HA BMC transplantation; AChRs in NMJs were labeled with tetramethylrhodamine-α-bungarotoxin (red, BTX); scale bar 10 µm. The results are means ± SD. * *p* < 0.05 vs. mdx (control).

An analysis of the structure of NMJs in *mdx* mice after BMC transplantation from C3HA mice under the aforementioned conditions revealed that in all treated groups of animals, a significant increase was observed in NMJs consisting of AChR clusters with the form of branches. However, the best results were achieved in the group of *mdx* mice irradiated with 3 Gy before transplantation and with the administration of Endoxan at a dose of 30 mg/kg twice after transplantation (Figure 6).

## 3. Discussion

This study investigated the effectiveness of the nonmyeloablative intravenous transplantation of BMCs in the structural recovery of the skeletal muscles of *mdx* mice. A comprehensive analysis of allogeneic and syngeneic BMC transplantation was performed during our experiments for inducing long-term effects.

To confirm the hypothesis that BMCs directly participate in muscle regeneration in *mdx* mice, we transplanted GFP-positive BMCs into *mdx* mice after X-ray irradiation. Six months later, we observed GFP-positive muscle cells and SMFs. Similar results have been described by other researchers during muscle fiber regeneration in response to injury [32,33]. However, there are not enough data in the literature concerning the direct participation of transplanted BMCs in SMF regeneration in muscular dystrophy in mdx mice.

Studies have attempted to treat DMD using BMC transplantation in *mdx* mouse models [23,34,35,36]. However, all such studies have failed to achieve a significant increase in the proportion of dystrophin-positive fibers. Most experiments have been conducted after total body irradiation by ionizing radiation at doses that have not allowed autologous bone marrow recovery (myeloablative transplantation). The author of [37] believed that the incorporation of normal nuclei into SMFs alone is not sufficient for activating the expression of muscle-specific genes. It is possible that the nonsignificant increase in dystrophin synthesis in *mdx* mouse SMFs after BMC transplantation following lethal irradiation is associated with the negative influence of ionizing radiation on muscle tissue. Muscle and nerve cells are highly differentiated and have low radiosensitivity; however, the negative effects of ionizing radiation on muscle tissue have been well documented. As the delayed necrosis of muscle was observed after therapeutic doses of radiation [41], it was suggested that exposure to 10 Gy of ionizing radiation is sufficient for inducing short-term muscle fatigue, cell death, and oxidative stress in the muscle [38].

In the current study, we used reduced doses of ionizing radiation (nonmyeloablative BMC transplantation). Nonmyeloablative allogeneic stem cell transplantation implies the use of nonmyeloablative conditioning (or reduced-intensity conditioning), which consists of low-dose chemo- and/or radiotherapy, thus decreasing regimen-related toxicity. A nonmyeloablative regimen will produce minimal cytopenia, and there is no need for stem cell support. Less intense regimens are generally better tolerated by patients who are normally excluded from conventional myeloablative transplantation [42]. The dose of X-ray radiation in nonmyeloablative BMC transplantation ranges from 0.5 to 3 Gy. The resulting chimerism after BMC transplantation into irradiated *mdx* mice suppresses the immune response of donor and recipient cells. The greatest effect was achieved after sublethal radiation at a dose of 2.7 Gy, which contributed to the long-term state of donor chimerism in the bone marrow of the recipient [43]. Our results revealed that X-ray irradiation at 3 Gy followed by syngeneic BMC transplantation led to a significant increase in dystrophin synthesis in *mdx* mouse muscles. The number of dystrophin-positive SMFs in the quadriceps femoris and diaphragm muscles had drastically increased 4 months after transplantation, reaching a maximum at 6 months and decreasing by 12 months after transplantation, but still remaining above the control level. A decrease in dystrophin synthesis in the 6-12-month interval after BMC transplantation may be associated with increased immunological conflict between dystrophin, which is presumably synthesized from donor mouse nuclei C57BL/6, and the immune cells of recipient mice (mdx) [44,45]. The period after BMC transplantation accounts for a significant part of an *mdx* mouse’s life, which indicates a long-term effect of transplantation.

We also observed that the percentage of SMFs without centrally located nuclei increased after BMC transplantation. The assessment of the percentage of muscle fibers with centrally located nuclei or, conversely, muscle fibers without centrally located nuclei has become a standard protocol for evaluating the effectiveness of drugs, and gene or cell therapy in DMD treatment [46]. It is suggested that reducing the number of muscle fibers with centrally located nuclei gives a better prognosis for DMD. When differentiating muscle fibers, the nuclei move from the central part of the fiber to the periphery; thus, mature muscle fibers do not have centrally located nuclei (Supplementary Figure S4). We assumed that the increase in the proportion of muscle fibers without centrally located nuclei represents an increase in the proportion of mature muscle fibers, which is characteristic of an intact muscle.

Complete muscle recovery is not possible without interaction with the nervous system and the formation of mature NMJs. The structures of NMJs are also impaired in *mdx* mice. Disorders mainly concern the postsynaptic part and are expressed in the breakdown of large AChRs into small islets [47,48,49,50,51,52,53]. Researchers have attributed the disturbance of the structure of NMJs in *mdx* mice to constant cycles of degeneration–regeneration [54,55], and not to the absence of dystrophin. A study found that in the extraocular muscles of *mdx* mice (rectus and oblique), in which there was no degeneration of muscle fibers despite the absence of dystrophin, NMJs retained a normal structure [54]. However, evidence suggests that dystrophin is directly involved in the organization of large aggregates of AChRs from small clusters during muscle regeneration, although it is not required for their initial clustering [47]. It can be assumed that the absence of dystrophin causes a disturbance of the structure of NMJs in *mdx* mice. This hypothesis has been verified in experiments performed on *mdx* and *dko* transgenic mice, in whose muscles various truncated dystrophin molecules were synthesized. Some truncated forms of dystrophin, such as microdystrophin^∆R4−R23^, have been demonstrated to be unable to eliminate synapse fragmentation in limb muscles despite preventing muscle degeneration [56,57]. One study also found that despite the large degree of muscle degeneration in *dko* mice, the degree of fragmentation of AChR clusters did not differ in *mdx* and *dko* mice; therefore, no direct correlation exists between the fragmentation of AChR clusters and muscle degeneration [58]. In our previous studies, we observed the restoration of electrophysiological properties and partial restoration of the structure of NMJs in the first 4 months after BMC transplantation, which coincided with an increase in the number of dystrophin-positive SMFs. Our previous studies have shown improvements in the electrophysiological properties of NMJs following cell therapy. Thus, the restoration of local hyperpolarization of the postsynaptic membrane was observed in mdx mice after irradiation at a dose of 3 Gy and subsequent transplantation of bone marrow cells to the level of normal mice C57BL/6 [59].

It was found that in C57Bl/6 mice, the value of the resting membrane potential (RMP) in the postsynaptic region of the membrane was, on average, −81.4 ± 0.5 mV. In mdx mice, the RMP was −74.5 ± 0.5 mV, which was significantly (*p* < 0.01) less than the RMP of the control muscles of C57Bl/6 mice. After cell therapy, the RMP significantly increased (*p* < 0.01) to −80.1 ± 0.5 mV, i.e., close to the level characteristic of control C57Bl/6 mice. The mean amplitude, rise time, and half-fall duration of the miniature end-plate potential (MEPPs) in mdx mice were significantly (*p* < 0.01) less than the control (C57BL/6), by 20, 25, and 20%, respectively. After cell therapy, the amplitude–time characteristics of MEPPs were returned to the control values [60]. This may indicate a connection between the structure and function of NMJs and the synthesis of dystrophin in muscle fibers [50,59,60]. The results of the present study indicated that the restoration of the structure of NMJs in *mdx* mice after BMC transplantation is preserved for 9 and 12 months after transplantation during the period where the number of dystrophin-positive SMFs decreases. It seems that the restoration of the structure of NMJs after cell therapy does not only depend on the level of dystrophin synthesis achieved in SMFs; rather, it has a more complex mechanism.

It is well known that to preserve the structure and improve the functional activity of SMFs, a certain level of dystrophin synthesis is required. However, in experiments in *mdx/utr−/−* mice, a study demonstrated that survival increased and motor function improved with a dystrophin level in the muscle fiber of less than 4%; furthermore, with a dystrophin level greater than 4%, the severity of histopathological signs also decreased and the expression of anti-inflammatory genes was preserved [61]. The authors of [61] believe that the level of dystrophin synthesis required to increase the life expectancy of patients with DMD may be lower than the level required to fully protect SMFs from damage. Our data indicate that despite the decrease in dystrophin synthesis 12 months after BMC transplantation, the number of NMJs with a normal structure was maintained at high levels and the number of dead SMFs did not increase. This finding supports the conclusion of van Putten et al. (2013), namely that a low level of dystrophin synthesis (4–5%) is sufficient for SMF survival [61]. In experiments on *mdx-Xist*^∆*hs*^ mice with congenitally low levels of dystrophin, a study found that the normal structure of NMJs was preserved in the presence of dystrophin in 19–50% of SMFs [62]. According to the calculations of Wells et al. (2019), effective DMD therapy can be achieved at a dystrophin synthesis level of 20% [63]. However, despite the low level of dystrophin revealed by our experiments 12 months post-transplantation, the proposed method could still be promising for increasing the life expectancy of patients with DMD. It is possible to use other methods to assess muscle structure and function in the future, for example, human described non-linear trimodal regression analysis (NTRA), which models the radiodensitometric distributions of X-ray computed tomography (CT) cross-sections. These methods make it possible to define 11 specific parameters of soft tissues, in particular muscles [64,65].

In our experiments, we used syngeneic BMCs from C57BL/6 mice for transplantation. Usually, for BMC transplantation, allogeneic donor materials are used. Therefore, in this study, we investigated how the synthesis of dystrophin and the structure of NMJs in *mdx* mice would change after the nonmyeloablative transplantation of allogeneic BMCs from C3HA mice. Cyclophosphamide (Endoxan) was chosen as the immunosuppressor. Studies have reported that the post-transplantation administration of cyclophosphamide induces transplantation tolerance in mice and is also considered a form of graft-versus-host disease prevention [66,67,68,69]. Allogeneic nonmyeloablative bone marrow transplantation with post-transplantation cyclophosphamide administration has also been applied in the treatment of nonmalignant hematological disorders [70,71]. Our results indicated that in the group of mice with Endoxan administration, the percentage of dystrophin-positive SMFs as well as that of SMFs without centrally located nuclei significantly increased, while the percentage of dead SMFs drastically decreased compared with *mdx* control mice. An analysis of NMJs revealed that in all groups of mice after allogeneic BMC transplantation, an increase occurred in the proportion of NMJs with AChR clusters in the form of branches, which is typical for normal mice.

Thus, our work demonstrated that the nonmyeloablative transplantation of BMCs from syngeneic or allogeneic donors promotes dystrophin synthesis and improves the structures of SMFs and NMJs of muscle tissue in *mdx* mice; thus, it can be considered a promising approach for treating DMD.

## 4. Materials and Methods

### 4.1. Animals

The study was performed on mutant mdx mice (males, age at transplantation: 2 months), which were gifted by Prof. T. A. Partridge (Hammersmith Hospital, London, UK). BMCs were isolated from male mice of the same age of the C57BL/6 and C3HA lines (obtained from the Rappolovo State Nursery, St. Petersburg, Russia) and transgenic GFP-expressing C57BL/6 mice (C57BL/6-Tg[ACT6EGFP]1Osb/J; donated by the Children’s Hospital Oakland Research Institute, Oakland, CA, USA). The mice were kept in the vivarium of the Institute of Cytology of the Russian Academy of Sciences on normal nutrition and under standard light conditions. All experimental procedures with animals were performed according to the Institutional Guidelines for the Care and Use of Laboratory Animals. All studies on animals were performed after receiving approval from the Institutional Animal Care and Use Committee of the Institute of Cytology RAS (assurance identification number F18–00380).

### 4.2. Bone Marrow Cell Isolation

BMCs from C57BL/6 and C3HA donor mice or transgenic GFP-expressing C57BL/6 mice were obtained similarly. Mice were sacrificed, the femur and tibia were isolated under sterile conditions, and bone marrow was washed out with sterile Dulbecco’s phosphate-buffered saline without Ca^2+^ and Mg^2+^ (DPBS Ca^−^, Mg^−^; BioloT, Saint-Petersburg, Russia). A single cell suspension was obtained by passing it through a syringe with a G27 needle.

### 4.3. Bone Marrow Cell Transplantation

BMC transplantation from C57BL/6 and C3HA donor mice or transgenic GFP-expressing C57BL/6 mice was performed similarly. mdx mice were irradiated on RAP-150/300-14 X-ray apparatus (Russia) at a dose of 2 or 3 Gy. One day after irradiation, the mice were injected with 0.2 mL (15–20 × 10^6^ nucleated BMCs) of BMC suspension in DPBS Ca^−^, Mg^−^ in the jugular vein.

After transplantation, the mice that were injected with BMCs from C3HA mice donors were additionally injected with the immunosuppressor Endoxan (cyclophosphamide; Baxter Oncology GmbH, Halle, Germany) either intraperitoneally or intramuscularly.

### 4.4. Muscle Collection

mdx mice (n = 3) injected with BMCs from transgenic GFP-expressing C57BL/6 mice donors were sacrificed 6 months after transplantation. mdx mice administered with BMCs from C57BL/6 mice donors were sacrificed at 2 (n = 4), 4 (n = 3), 6 (n = 3), 9 (n = 6), and 12 (n = 8) months after transplantation. mdx mice injected with BMCs from C3HA mice donors were sacrificed 6 months after transplantation. Nontransplanted mdx mice and C57BL/6 mice of the same age were used as controls.

The quadriceps femoris and diaphragm muscles from all mdx mice were isolated and precooled in liquid nitrogen. Frozen sections (10 µm thick) were cut from the quadriceps femoris and diaphragm using a Bright Co Ltd. (London, UK) cryostat. After drying, sections were fixed in a mixture of ethanol and methanol (1:1) for 1 min for immunohistochemistry or 2 min in ethanol at −20 °C for the other staining.

### 4.5. Assessment of Presence of GFP-Positive Cells and SMFs in Muscles after Transplantation of BMCs from Transgenic GFP-Expressing C57BL/6 Mice Donors

Slides with quadriceps femoris sections from mdx mice after the transplantation of BMCs from transgenic GFP-expressing C57BL/6 mice were stained with propidium iodide for 5 min, washed for 5 min in phosphate-buffered saline (PBS; Biolot, Saint-Petersburg, Russia), and mounted in Fluoromount-G (SouthernBiotech, Birmingham, AL, USA). Digital images of muscle sections were obtained with a Leica TCS SL confocal microscope (Leica Microsystems, Wetzlar, Germany).

### 4.6. Immunohistochemistry

To detect dystrophin, slides with quadriceps femoris and diaphragm sections were incubated in 3% bovine serum albumin (Sigma, Saint Louis, MO, USA) for 30 min and washed for 5 min in PBS (Biolot, Saint-Petersburg, Russia); then, polyclonal rabbit antibodies to dystrophin (Abcam, Cambridge, MA, USA) at a 1:100 dilution were added and incubated at +4 °C overnight. After 3-fold washing for 5 min in PBS, the slides were incubated in 0.03% H_2_O_2_ solution for 30 min and washed in PBS 3 times for 5 min; then, Avidin/Biotin Blocking Kit reagents (Zymed Laboratories Inc., Invitrogen, South San Francisco, CA, USA) were added to reduce the nonspecific binding of streptavidin with endogenous biotin according to the manufacturer’s instructions. Next, the slides were washed 3 times for 5 min in PBS, and secondary antibodies to rabbit immunoglobulins labeled with biotin (Sigma, Saint Louis, MO, USA) at a 1:100 dilution were added and incubated for 1 h. Subsequently, the slides with sections were washed in PBS 3 times for 5 min and peroxidase-conjugated streptavidin was added at a 1:150 dilution (Sigma, Saint Louis, MO, USA) before being incubated for 30 min, followed by washing in PBS 3 times for 5 min; then, the slides were incubated with diaminobenzidine (10 mg of diaminobenzidine was dissolved in 10 mL of PBS and mixed with 0.1 mL of 1% H_2_O_2_ solution in PBS) for 5 min and washed in water. If staining was not sufficiently saturated, the slides were further incubated in diaminobenzidine with cobalt acetate for 5–20 min and then washed in running water. The nuclei were stained with Giemsa’s staining solution, and then the sections were dehydrated with graded alcohol, immersed in 3 portions of xylene, and mounted in Canada balsam. For the nonspecific binding of secondary antibodies, slides with sections without primary antibodies were used as a control.

In dystrophin-stained sections, the number of dystrophin-positive SMFs, total number of SMFs, and percentage of dystrophin-positive SMFs in the direct muscle were counted. The percentage of SMFs that did not contain centrally located nuclei was determined to assess the differentiation of myotubes into muscular fibers. This was used as an indicator of the differentiation of SMFs. The percentage of SMFs without central nuclei as well as the proportion of dead SMFs was conducted in histological sections using a commonly accepted method.

### 4.7. The Number of Dead SMFs Evaluation

The slides with quadriceps femoris and diaphragm sections were stained with H&E according to the generally accepted method. The number of SMFs that were in the necrosis region (determined by morphological characteristics) and the total number of SMFs in the direct muscle were counted. The percentage of dead SMFs was calculated.

### 4.8. Masson’s Trichrome Staining

Trichrome staining was performed using a Masson Staining Kit (Biovitrum, Saint-Petersburg, Russia) for the visualization of connective tissue.

### 4.9. Investigation of the Neuromuscular Junctions’ Structure

Diaphragms from experimental animals were dissected and placed in 5% formalin (Biovitrum, Saint-Petersburg, Russia) for 1 h at +4 °C and then moved to 0.5% formalin, incubated overnight at +4 °C, and washed for 30 min in PBS. Next, diaphragms were incubated in tetramethylrhodamine-α-bungarotoxin (TMR-α-BTX; Biotium, Fremont, CA, USA) at a concentration of 1 μg/mL for 1 h, then washed in PBS 3 times for 5 min, and mounted in Fluoromount-G (SouthernBiotech, Birmingham, AL, USA). Digital images of neuromuscular junctions (NMJs) from whole-mount tissue were obtained with a Leica TCS SP5 confocal microscope (Leica Microsystems, Wetzlar, Germany). In the obtained images of each NMJ, a maximum-intensity flat plane z projection was made from Z-stacked images. The shape of the acetylcholine receptor clusters (AChRs) consisted of this NMJ, and extended branches or individual islets were determined. Using ImageJ 1.53c software (NIH, Bethesda, MD, USA), the area of each AChR cluster, the number of such clusters, the total area of the NMJ, the total stained area (the sum of the areas of all clusters constituting the NMJ), and the dispersion index (DI; calculated as the ratio of the total stained area of the NMJ to the total area of NMJ multiplied by 100) were quantified.

### 4.10. Statistical Analysis

Statistical analysis was performed using GraphPad Prism 9 (Boston, MA, USA). The data are presented as mean ± SEM. We used Student’s *t* test for the data analysis. Differences were considered statistically significant when *p* < 0.05.

## Data Availability

All data are included in the manuscript and Supplementary.

## Supplementary Materials

The supporting information can be downloaded at: https://www.mdpi.com/article/10.3390/ijms24108892/s1.
