# Supplementary material for: Accumulation of Dystrophin-Positive Muscle Fibers and Improvement of Neuromuscular Junctions in mdx Mouse Muscles after Bone Marrow Transplantation under Different Conditions
Source: Int J Mol Sci. 2023 May 17;24(10):8892. doi: 10.3390/ijms24108892 (PMC10219484; doi:10.3390/ijms24108892)
Supplement: Supplementary file 1 [file ijms-24-08892-s001.zip › ijms-2358116-supplementary.pdf]

Supplementary.

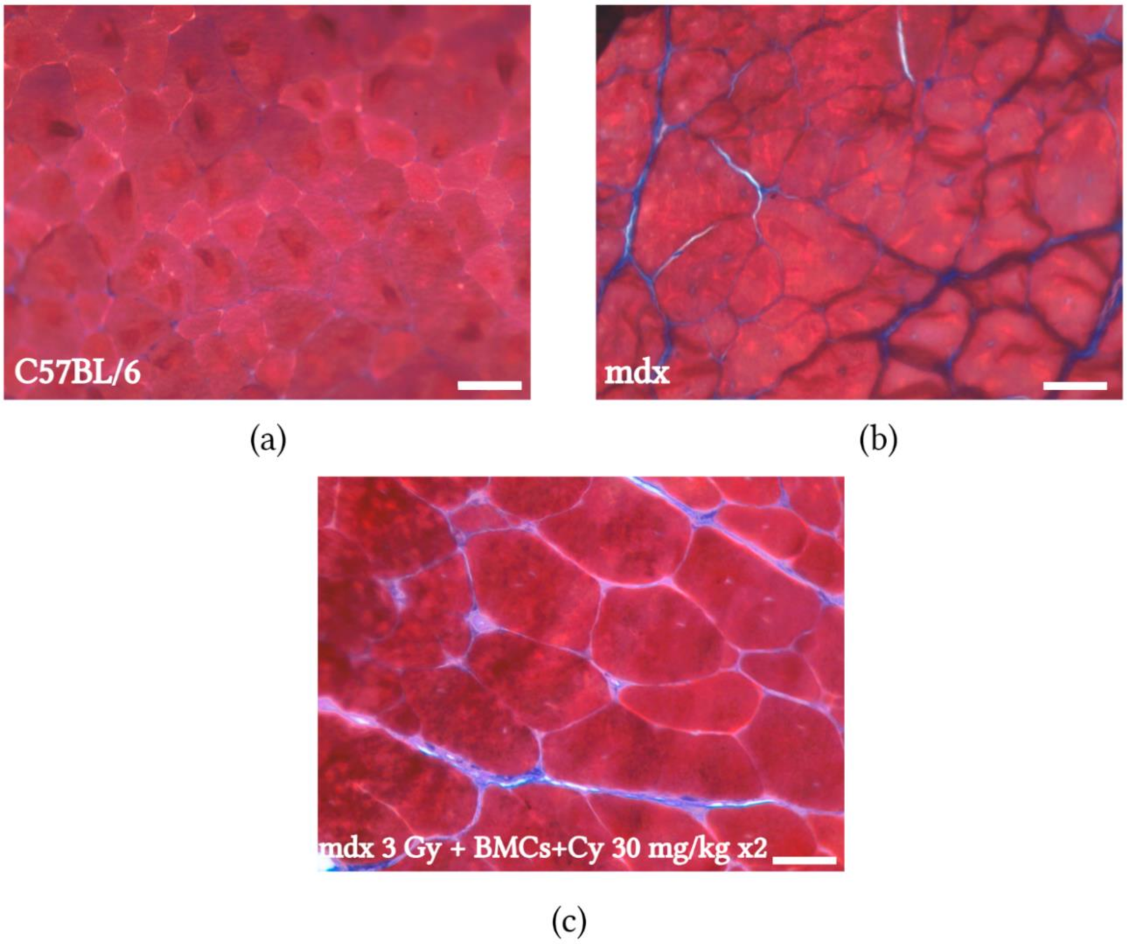

Figure S1. The fibrosis level evaluation in the quadriceps femoris after BMC transplantation. (a) Masson`s trichrome staining of the quadriceps femoris of normal *C57BL/6* mice; scale bar 50 µm; (b) Masson`s trichrome staining of the quadriceps femoris of *mdx* mice; fibrosis (blue); scale bar 50 µm; and (c) Masson`s trichrome staining of the quadriceps femoris of *mdx* mice after BMCs transplantation; the decreasing part of fibrosis in muscle (blue); scale bar 50 µm.

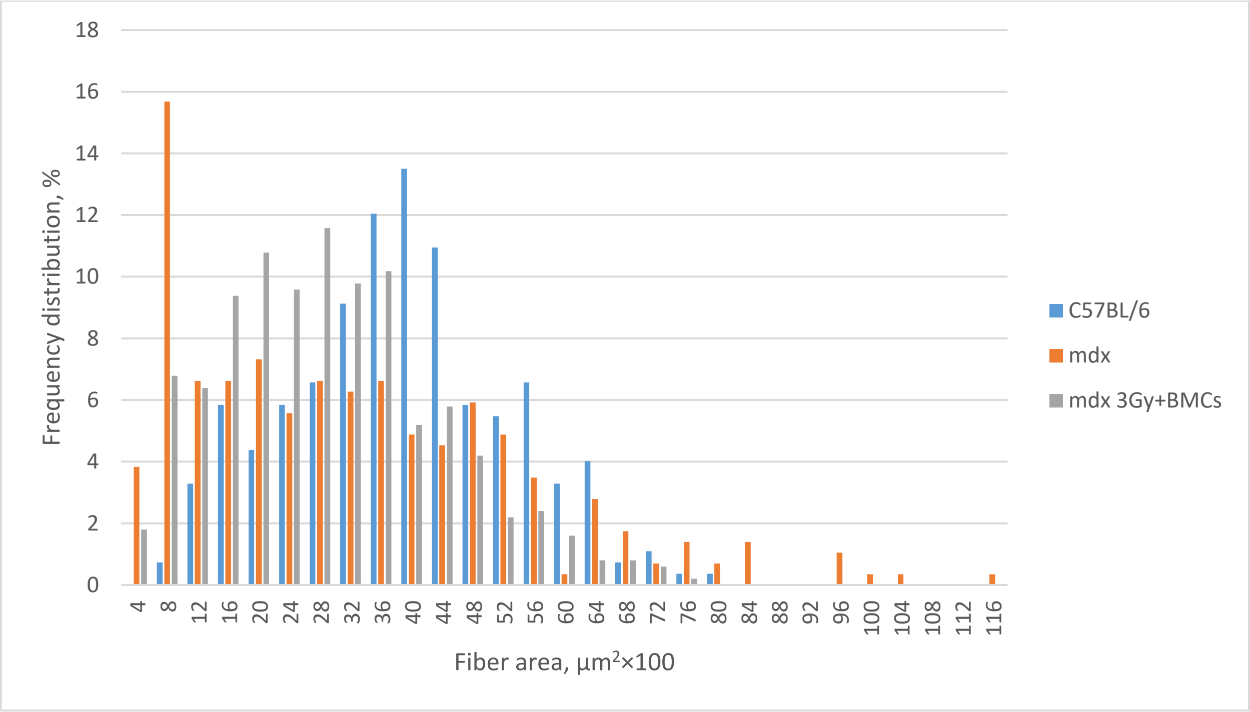

Figure S2. The muscle fiber cross sectional area frequency distribution.

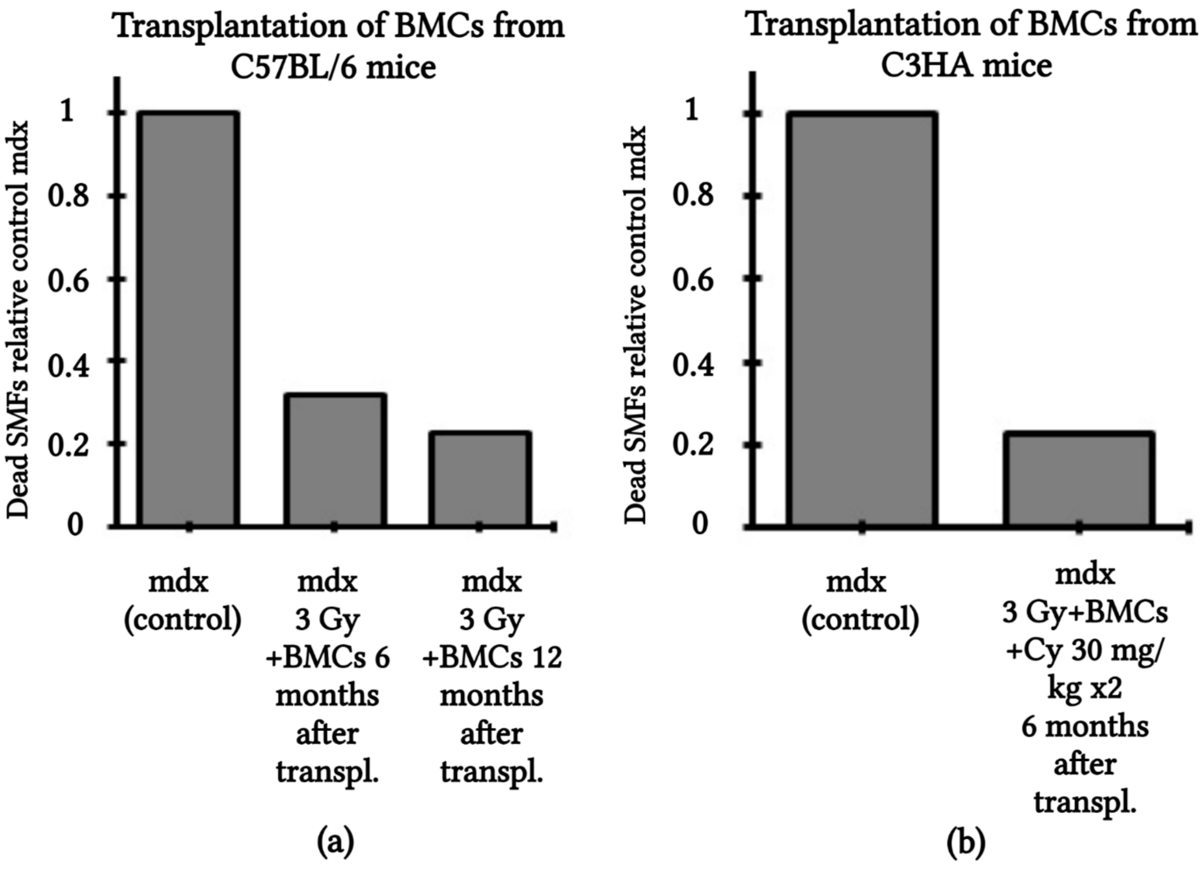

Figure S3. Reduction in the number of dead SMFs after BMC transplantation. (a) Decrease in the number of dead SMFs after BMC transplantation from C57BL/6 mice; (b) Decrease in the number of dead SMFs after BMC transplantation from C3HA mice.

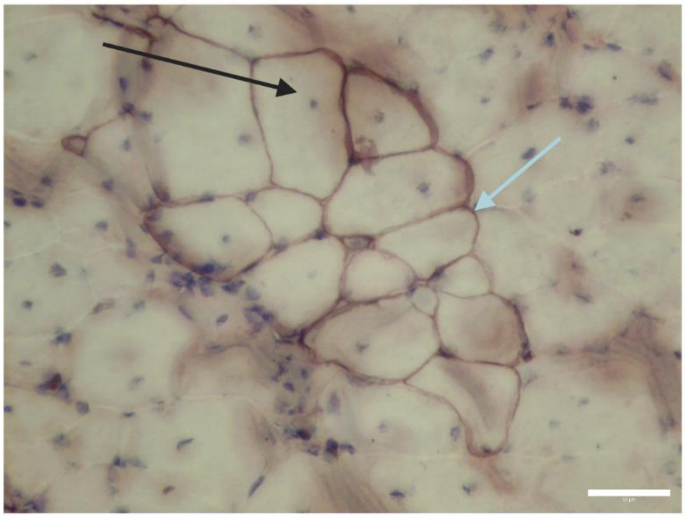

Figure S4. Immunohistochemical staining of SMFs from the quadriceps femoris of *mdx* mice after C3HA BMC transplantation; focus of dystrophin-positive SMFs were observed. Dystrophin-positive SMFs with (black arrow) and without (blue arrow) centrally located nuclei; scale bar 50 μm.
